# Methylene Blue-Loaded Liposomal Nanocarriers Enhance the Efficacy of Photodynamic Therapy against *Candida auris* Biofilm

**DOI:** 10.1021/acsinfecdis.5c00941

**Published:** 2025-12-25

**Authors:** Patricia Michelle Nagai de Lima, Akram Abbasi, Veronica LaMastro, Juliana Campos Junqueira, Anita Shukla

**Affiliations:** † Center for Biomedical Engineering, School of Engineering, 6752 Brown University, Providence, Rhode Island 02912, United States; ‡ Institute of Science and Technology, 327019 São Paulo State University (UNESP), São José dos Campos, São Paulo 12245-000, Brazil

**Keywords:** Candidiasis, Candida auris, photodynamic therapy, methylene blue, liposomes, biofilms

## Abstract

*Candida auris* poses a significant healthcare challenge, particularly within immunosuppressed patients. This pathogen can colonize the skin and develop biofilms associated with increased antifungal drug resistance that are difficult to treat with a limited antifungal repertoire. Some adjuvant treatments have been investigated, such as photodynamic therapy (PDT), which employs a photosensitizer (PS) irradiated by light. However, most PSs available suffer from poor biofilm penetration. In this in vitro study, a nanocarrier system was proposed as a possible strategy to facilitate the methylene blue (MB) photosensitizer penetration into biofilm and improve PDT action against *C. auris*. For this, positively (MB-P) and negatively (MB-N) charged liposomes encapsulating MB were successfully fabricated. In the PDT results, both liposome formulations eradicated planktonic cells of *C. auris* at minimum fungicidal concentrations (MFC) equivalent to those of free MB. MB-loaded liposomes showed enhanced penetration within biofilms and reduced *C. auris* biofilm burden ∼2× more compared to free MB. Additionally, biofilm biomass was reduced up to 37% with MB-loaded liposomes, while free MB only achieved ∼3% reduction. Furthermore, PDT mediated by MB-P or MB-N led to the production of reactive oxygen species (ROS) 2× higher than free MB, leading to greater oxidative damage toward *C. auris* biofilms. Finally, the biocompatibility of MB-loaded liposomes was examined against mammalian fibroblasts; MB-loaded liposomes maintained ∼80% cell viability compared to ∼58% viability for free MB. Promisingly, MB-P and MB-N liposomes were able to enhance the in vitro activity of PDT on *C. auris* biofilms, inciting the development of in vivo studies to validate their efficacy and safety.

## Graphical abstract

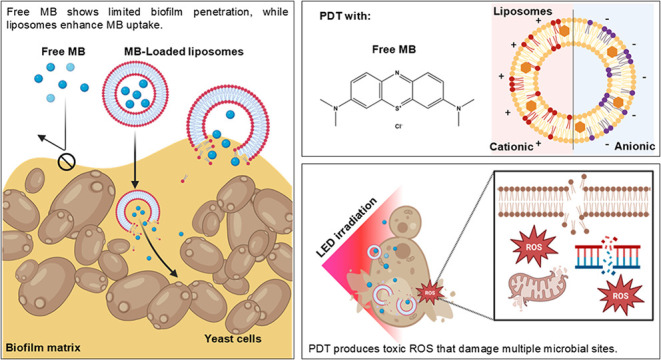

*Candida auris* is an opportunistic fungal pathogen that easily colonizes the skin of hospitalized patients and can enter the bloodstream through a wound or an inserted medical device, leading to invasive and fatal infections.^1^ *C. auris* represents a global threat due to the rapid development of resistance toward antifungal agents and disinfectants.^2–4^ Multidrug- and pan-resistant strains of *C. auris* have been identified globally, making treatment challenging.^5,6^ Due to this concerning resistance pattern, *C. auris* was ranked in the critical priority group on the Fungal Priority Pathogen List released by the World Health Organization (WHO).^7^ Additionally, the Centers for Disease Control and Prevention (CDC) identified *C. auris* as an urgent threat in the 2019 Antibiotic Resistance Threats Report.^8^

The formation of *C. auris* biofilms, a community of fungal cells surrounded by a secreted extracellular matrix, further complicates infection treatment. The biofilm structure limits immune cell infiltration and antifungal drug penetration. Cells within the biofilm can also exhibit antifungal drug resistance due to increased expression of drug efflux pumps.^9–11^ With resistance toward an already limited antifungal drug repertoire (∼20 Food and Drug Administration (FDA) approved),^4,12^ alternative treatments such as photodynamic therapy (PDT) are of great interest to treat multidrug-resistant-related biofilm infections.

PDT is a therapeutic approach that involves light activation of a photosensitizer (PS), resulting in localized cell death through the generation of cytotoxic reactive oxygen species (ROS).^13^ Due to the rapid generation of ROS following light exposure and broad cell-targeting potential of PDT, the development of fungal resistance to this treatment is highly unlikely,^14,15^ providing a promising strategy for the treatment of *C. auris* biofilm infections. However, the limited penetration of PSs within biofilm structures can impair the efficacy of PDT.^16,17^

In this context, nanocarrier systems have been employed, such as liposomal formulations.^17,18^ Liposomes are lipid-based nanoparticles primarily composed of a phospholipid bilayer with amphiphilic properties and can encapsulate both hydrophilic and hydrophobic drugs.^19,20^ The functionality of liposomes can be modulated through the optimization of lipid composition, the incorporation of stimuli-responsive compounds, and the use of ligand-specific receptors to stimulate targeted delivery. These modifications enhance their overall performance and functionality for pharmaceutical purposes,^19,21,22^ including improving drug stability and reducing drug toxicity.^23^ In addition, the use of liposomes as a drug delivery system can lead to enhanced drug penetration into cell membranes through fusion, passive transport, or electrostatic interaction.^24,25^ However, liposome interaction with fungal cells is little understood.

Recent studies have explored a range of liposomal formulations to encapsulate different PSs. For instance, zinc phthalocyanine (ZnPC) in liposomes has demonstrated enhanced cytotoxicity of PDT against oral and pharyngeal carcinoma cells. These liposomes led to the effective delivery of ZnPC, improving its biodistribution and therapeutic impact.^26^ Another PS, chlorine e6 (Ce6), was encapsulated in cationic liposomes, which significantly enhanced the antifungal PDT effect against both susceptible and drug-resistant clinical isolates of *Candida albicans*.^27^ Moreover, the encapsulation of Verteporfin (PS used in the treatment of macular degeneration) in liposomes improved its bioavailability by preventing self-aggregation and ensuring stability, leading to the FDA approval of the photodynamic drug Visudyne.^18,28^

However, while the advancements of PDT using liposomes are significant, certain limitations persist. Many current liposomal formulations face challenges such as limited tissue penetration, suboptimal PS release rates, and issues related to maintaining stability and effective light penetration.^29^ Aiming to improve the treatment of *C. auris* biofilm-related infections, we fabricated two different liposomal formulations containing methylene blue (MB). To investigate the delivery of MB to *C. auris* through the liposomes obtained, we assessed their interaction with *C. auris* cells in both planktonic and biofilm states. We then examined the antifungal efficacy of PDT using both MB-loaded liposomes against planktonic cells and biofilms of *C. auris*, as well as the production of ROS. Finally, we assessed the cytocompatibility of these liposome formulations in fibroblasts, a common cellular component of the skin. Overall, the MB-loaded liposomes developed here enhanced penetration within *C. auris* biofilms and increased antifungal activity compared to free MB, with minimal cytotoxicity for mammalian cells.

## Results and Discussion

In this study, we integrated principles from microbiology, nanotechnology, and phototherapy for designing liposome-based nanoparticles targeted to PDT. This interdisciplinary approach allowed us to optimize the biocompatibility and customizable properties of liposomes^21,30^ to enhance the PS delivery within microbial biofilms. MB alone suffers from poor solubility and limited penetration into biofilms, as well as potential toxicity at high concentrations.^31,32^ In general, liposomes can mitigate these issues by improving the solubility within biofilms and reducing the toxicity of MB, providing a more effective treatment.^18,33–35^ Studies have shown that liposomes can fuse with biofilm components of various types of bacterial and fungal biofilms, including those formed by *Candida* spp.^21,35–38^ However, little information is given about *C. auris*. In the context of PDT, while this is the first study to evaluate the PS-loaded liposomes on *C. auris* biofilms, related photosensitizer nanocarrier approaches have shown efficacy against *C. albicans* biofilms.^27^ The novelty of this study lies in combining the MB photosensitizer with liposomal formulations to control specifically the biofilms formed by *C. auris*.

### MB-Loaded Liposome Fabrication and Characterization

Assessing the properties of liposomes is crucial to optimizing their efficacy as drug delivery vehicles. Key parameters, such as size and surface charge, play pivotal roles in determining their performance. Size affects biodistribution and cellular uptake, with smaller liposomes typically exhibiting better tissue penetration and clearance rates. Charge can influence stability and interactions with biological membranes, affecting circulation time and targeting efficiency.^18,34^

In this study, two liposomal formulations were fabricated using a thin film hydration method, followed by extrusion. DPPC, a saturated, zwitterionic lipid, was utilized as the main lipid in both formulations, as previous studies have shown good biocompatibility in therapeutic applications containing this lipid^39^ and previous liposome formulations containing DPPC have been used to encapsulate MB.^32,40^ Unsaturated phospholipids should be avoided for PDT use because they are highly susceptible to lipid peroxidation, making them well-known targets for ROS.^32^ The lipid dimethyldioctadecylammonium bromide (DDAB) was added to formulations to generate positively charged liposomes (PL), while DPPG was added to generate negatively charged liposomes (NL).

After fabrication, the liposomes obtained were characterized based on their physicochemical properties related to hydrodynamic diameter, polydispersity index (PDI), and ζ-potential. The parameters of MB encapsulation efficiency (EE), MB drug loading (DL), and stability were also determined.

More specifically, we verified that NL and PL formulations exhibited hydrodynamic diameters of ∼156 and ∼172 nm, respectively. Upon the addition of MB, the diameter of each liposome formulation increased; NL containing MB (MB-N) exhibited hydrodynamic diameters ∼55 nm greater compared to blank NL formulations (∼24% increase), and PL containing MB (MB-P) formulation increased ∼32 nm compared to their blank counterpart (∼16% increase) (Figure 1A). Similarly, Leo et al.^41^ observed an 18.9% increase in the diameter of their liposomes after encapsulating MB. Thus, this increase in hydrodynamic diameter suggests the successful encapsulation of MB. All liposome formulations exhibited PDI values of <0.2, indicating minimal variation in the hydrodynamic diameters (Figure 1B). In drug delivery applications using lipid-based carriers such as liposomes, a PDI of less than 0.2 ensures homogeneity of the vesicles, indicating a uniform particle size distribution.^42^ In relation to their electrostatic properties according to ζ-potential measurements, PL and NL formulations were positively and negatively charged, respectively, due to the charges of DDAB and DPPG (Figure S1). The addition of MB increased the ζ-potential from +24.3 to +27.8 mV for MB-P, while MB-N liposomes exhibited a decrease in magnitude (−21.8 mV) compared to NL formulations (−26.6 mV), most likely due to the cationic nature of MB (Figure 1C).

**Figure 1:**
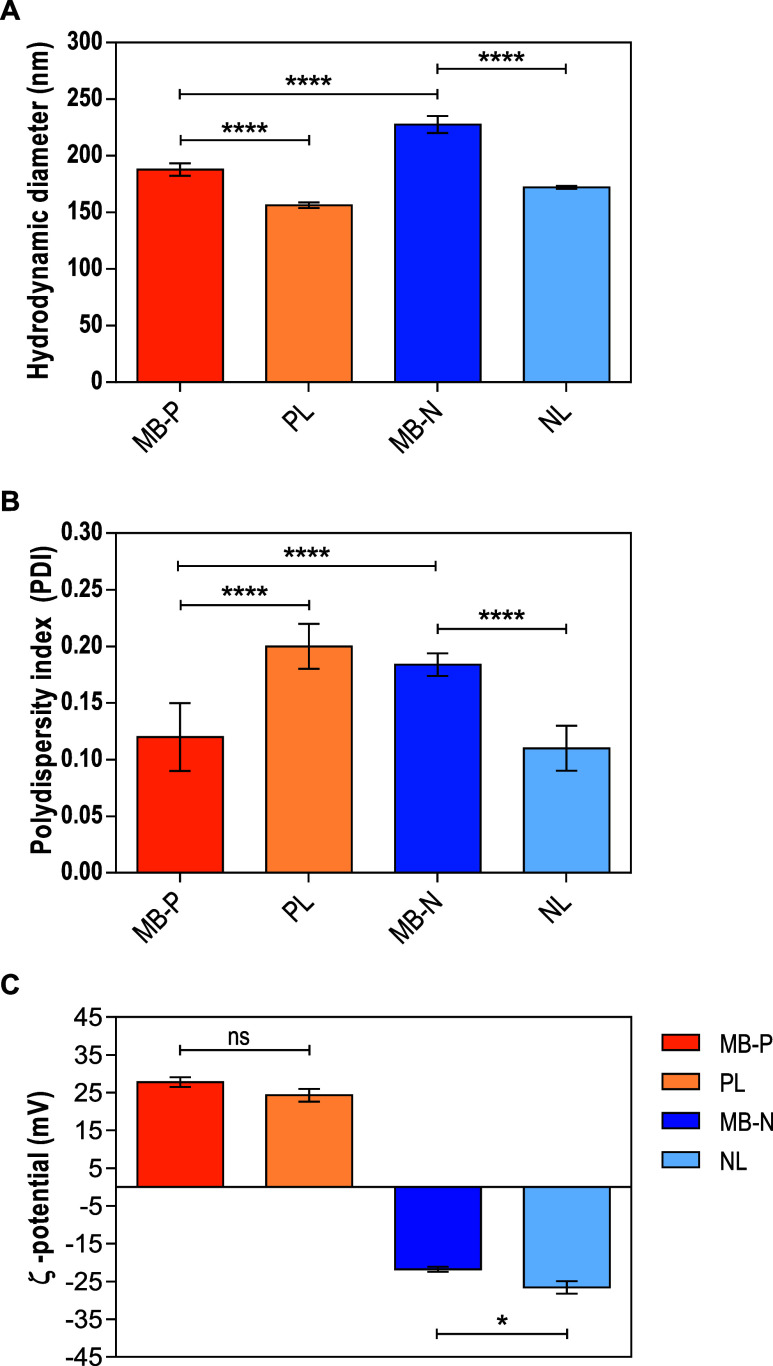
Physicochemical properties of liposomal formulations were analyzed in phosphate-buffered saline (PBS) at pH 7.4 using dynamic light scattering (DLS). (A) Hydrodynamic diameters. (B) Polydispersity index PDI. (C) ζ-Potential. MB-P: positively charged liposomes encapsulating methylene blue (MB); MB-N: negatively charged liposomes encapsulating methylene blue (MB); PL: positively charged liposomes; NL: negatively charged liposomes. Results are reported as mean ± standard deviation (*n* = 3).

Certainly, the physicochemical properties of liposomal formulations had influence on the parameters of MB encapsulation efficiency (EE) and MB drug loading (DL), the results of which are presented in Table 1. The cationic nature of MB^32^ suggests that the higher EE and DL values observed with MB-N liposomes may have resulted from electrostatic interactions between MB and the negatively charged liposome surfaces. Additionally, the increased hydrodynamic diameter of the MB-N formulations compared to blank negative liposomes (NL) supports the hypothesis that a greater quantity of MB was encapsulated within these liposomes. This finding underscores the potential of using negatively charged liposomal carriers for the effective delivery of cationic PSs. For instance, MB-P which is composed of DPPC:DDAB:CHOL can be compared to those reported by Boccalini et al.,^32^ who observed an EE of ∼8% and a DL of ∼9% in a liposome composition DPPC:CHOL: dioleyldimethylammonium chloride, whereas Wu et al.,^13^ observed a slightly lower EE (∼5%) in a neutral charged liposome composed of DPPC:poly­(carboxybetaine) modified 1,2-dipalmitoyl-*sn*-glycero-3-phosphoethanolamine. However, direct comparisons of these results with our formulation are limited by differences in liposome composition, initial MB concentration, and lipid-to-drug ratio across studies.

**Table 1:** Mean and Standard Deviation from the Results of MB Encapsulation Efficiency (EE) and MB Drug Loading (DL)^a^

sample	MB encapsulation efficiency (%)	MB drug loading (%)
MB-P	10.75 ± 0.91	5.07 ± 0.52
MB-N	14.41 ± 1.25	5.94 ± 0.98

a MB-P: positively charged liposomes encapsulating methylene blue (MB); MB-N: negatively charged liposomes encapsulating MB; PL: positively charged liposomes; NL: negatively charged liposomes.

Finally, stability was evaluated by monitoring hydrodynamic diameter, PDI, and ζ-potential of the liposomes on the day of preparation and after 30 and 60 days of storage at 4 °C in dark conditions. The formulations maintained their physicochemical properties up to 30 days, after which instability was observed (Figure 2). Thus, the current formulations are suitable for use within this time frame but require optimization for longer-term storage.

**Figure 2:**
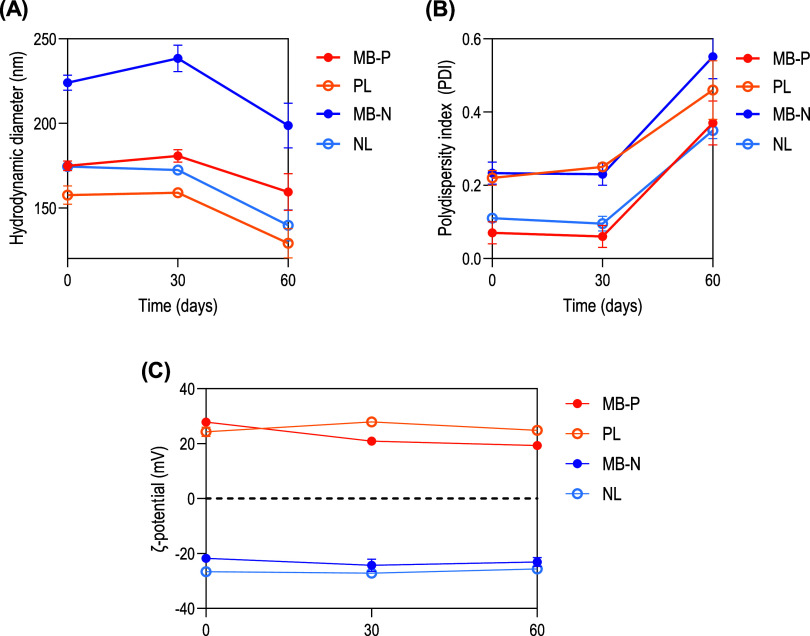
Stability of liposomal formulations evaluated at the day of fabrication (0 days) and after 30 and 60 days of storage at 4 °C. Measurements were performed in 1× PBS at pH 7.4 using dynamic light scattering. (A) Hydrodynamic diameter. (B) Polydispersity index (PDI). (C) ζ-Potential. MB-P: positively charged liposomes encapsulating methylene blue (MB); MB-N: negatively charged liposomes encapsulating MB; PL: positively charged liposomes; NL: negatively charged liposomes. Results are reported as mean ± standard deviation (*n* = 3).

Although various physicochemical properties of MB-loaded liposomes were evaluated in this study, a full characterization is still required to consider them as an effective photosensitizer delivery system for PDT. For this, several analytical methods proposed by regulatory agencies, such as European Medicines Agency (EMA) and US Food and Drug Administration (FDA), must be employed in the next studies to determine the following properties: morphology by transmission electron microscopy (TEM), drug release in physiologically relevant media, liposome phase transition temperature, and liposome integrity in different salt concentration, pH, and temperature.^43^

### Interaction of Liposomal Formulations with *C. auris* Cells in Planktonic and Biofilm Stages

With the successful fabrication of MB-loaded liposomes, we then evaluated their interaction with *C. auris* cells in planktonic and biofilm stages. Given the negatively charged surface of fungal cells,^44^ we hypothesized that the cationic MB-P liposomes would exhibit greater interaction with *C. auris* compared to MB-N liposomes. This hypothesis is based on previous studies demonstrating that positively charged nanoparticles interact more effectively with negatively charged fungal and mammalian cells due to electrostatic attraction.^34^ Evaluating the interaction between liposomes and their target is crucial for optimizing drug delivery systems, as effective interaction ensures targeted delivery and minimizes off-target toxicity.^20,45^

Our study about liposome interaction with *C. auris* cells in the planktonic state was assessed using flow cytometry, where the percentage of live fungal cells expressing MB fluorescence was quantified as an indication of fungal cell-liposome interaction. The flow cytometry gating strategy is outlined in the Supporting Information (Figure S2). We verified that ∼66 and ∼61% of live *C. auris* cells expressed MB following incubation with MB-P and MB-N formulations, respectively (Figure 3A). Although MB-P showed a slightly higher interaction with fungal cells, there was no statistical difference between the two formulations. The liposomes and planktonic cell interaction was also confirmed by confocal laser scanning microscopy (CLSM) analysis. MB were found in *C. auris* cells treated with both MB-P and MB-N formulations, localized predominantly in the cell wall of the yeast cells. However, when quantifying the MB fluorescence intensity in CLSM images, no statistical difference was observed between cells treated with either MB-P or MB-N (Figure 3B,C).

**Figure 3:**
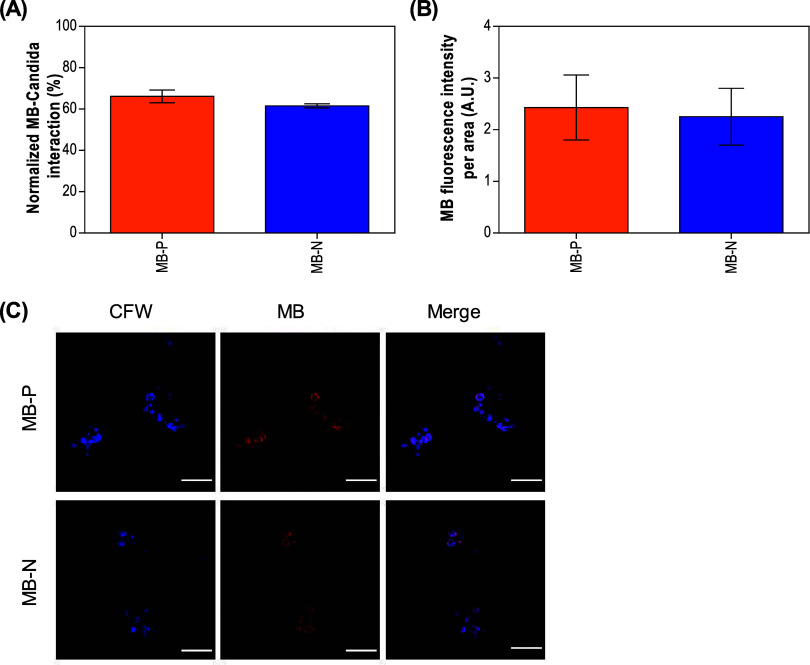
Interaction of MB-loaded liposome formulations with planktonic cells of *C. auris* B11220. (A) The percentage of live *C. auris* cells positive for MB fluorescence after 15 min of incubation with MB-loaded liposomes measured via flow cytometry. Data are reported as mean ± standard deviations (*t* test two-tailed analysis, *n* = 3, α = 0.05). (B) MB fluorescence intensity per area after 15 min of incubation with MB-loaded liposomes measured from CLSM images. Data are reported as mean ± standard deviations (two-tailed *t* test, *n* = 12, α = 0.05). (C) Representative confocal images of *C. auris* after 15 min of incubation with MB-P and MB-N formulations. *C. auris* cells were stained with calcofluor white (CFW) (blue), while MB is shown in red. Fungal cells displaying red fluorescence or a purple hue suggest fungal cell-liposome interaction. Scale bar: 20 μm. MB-P: positively charged liposomes encapsulating methylene blue (MB); MB-N: negatively charged liposomes encapsulating methylene blue (MB).

In a study conducted by Yang et al., cationic liposomes composed of dimyristoyl-*sn*-glycero-phosphatidylcholine (DMPC), cetyltrimethylammonium bromide (CTAB), and the PS chlorine e6 (Ce6) had a significantly increased binding to the planktonic form of *C. albicans* compared to negatively charged liposomes containing DMPC only. These results of Yang et al. can be attributed to the liposome size, as their cationic liposomes were smaller (80 nm) compared to our cationic liposomes (187 nm). It is known that smaller liposomes have a higher surface-area-to-volume ratio, potentially enhancing the interfacial interactions with the cell wall. In addition to liposome sizes, the differences in the fungal species can also play a significant role. Yang et al. focused on *C. albicans*, whereas our study involved *C. auris*. Both species have cell walls composed of mannan, β-glucan, and chitin. However, *C. auris* has a thicker outer mannan layer, longer mannoprotein fibrils, a lower proportion of β-glucan, and lack of phosphomannan.^46^ *C. auris* is known for its unique cell wall properties and higher resistance to antifungal agents compared to *C. albicans*. These differences might influence the efficacy of liposomes in enhancing cellular uptake in *C. auris* and *C. albicans* species. This highlights the interplay between the type of photosensitizer, the physicochemical properties of liposomal formulations, and the specific characteristics of the target fungal species as crucial factors in determining the success of PSs delivery to *Candida* cells.

In our study about liposome interaction with *C. auris* cells in the biofilm state, we used CLSM analysis. The obtained images showed the enhanced MB delivery for both liposome formulations when compared with free MB, with statistically significant differences in the values of MB fluorescence intensity (Figure 4). Additionally, MB-P liposomes had a greater MB signal within *C. auris* biofilms compared to MB-N formulations, potentially due to enhanced electrostatic interaction between the *C. auris* biofilms and positively charged liposomes. Recent studies have also demonstrated the effectiveness of positively charged nanoformulations against *C. auris* biofilms. Jaromin et al.^47^ studied a novel antifungal compound (PQA-Az-13) encapsulated in cationic liposomes. Their liposomal formulation exhibited strong activity against *C. auris* biofilms in both in vitro and ex vivo skin colonization models. In another study conducted by Lara et al.,^48^ cationic AgNPs effectively inhibited *C. auris* biofilm, with IC_50_ values of 0.06 ppm for biofilm formation and 0.48 ppm for preformed biofilms. Moreover, AgNP-functionalized surfaces, such as silicone elastomers and bandage dressings, exhibited over 50% biofilm inhibition and retained efficacy after multiple washes, highlighting their potential in preventing *C. auris* infection.

**Figure 4:**
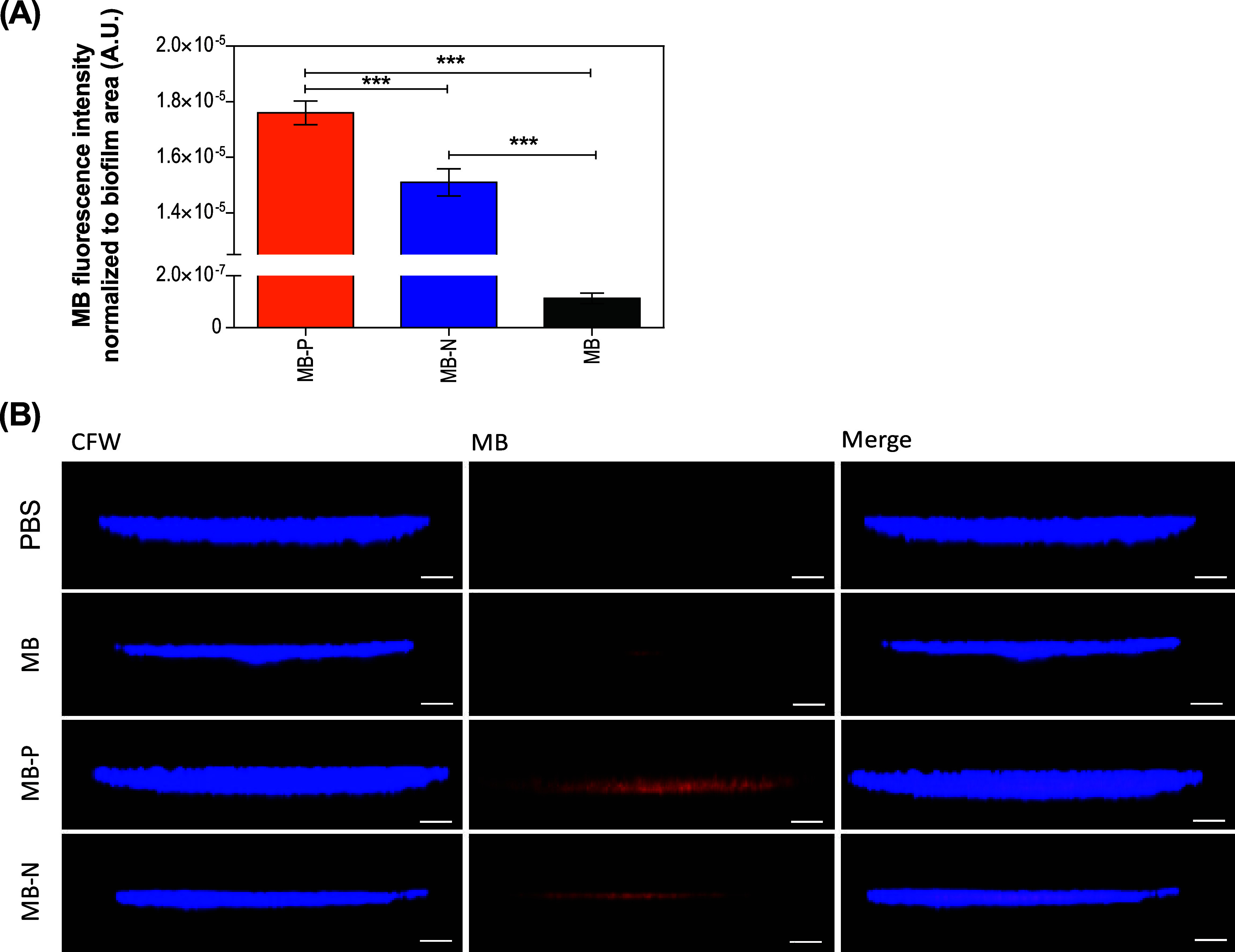
Interaction of MB-loaded liposome formulations with biofilms of *C. auris* B11220. (A) Corresponding MB fluorescence intensity in *C. auris* biofilms evaluated by CLSM, where higher fluorescence intensity indicated greater *C. auris* cell-liposome interaction and MB delivery within biofilms. Data are reported as mean ± standard deviations (one-way analysis of variance (ANOVA) with Tukey’s posthoc analysis, ***p ≤ 0.001, *n* = 3, α = 0.05). (B) Representative confocal images of *C. auris* B11220 biofilm after 15 min incubation with PBS only, free MB, MB-N, and MB-P. *C. auris* cells were stained with CFW (blue), while MB is shown in red. Fungal cells displaying red fluorescence or a purple hue suggest interaction with liposomes. Scale bar: 50 μm. PBS: phosphate-buffered saline; PL: positively charged liposomes; NL: negatively charged liposomes; MB: Methylene blue; MB-P: positively charged liposomes encapsulating MB; MB-N: negatively charged liposomes encapsulating MB.

Taken together, our results showed that both liposomal formulations were able to interact with planktonic cells and the biofilms of *C. auris*. However, only in the biofilm state did MB-P liposomes have a greater interaction with *C. auris* than MB-N liposomes. These results may be attributed to the distinct surface protein expression profiles of *Candida* cells in the planktonic or biofilm stages. Cells of *C. auris* in biofilms overexpress several proteins, such as orthologs of *C. albicans* Spe3p, Tdh3p, Sod2p, Ywp1p, and Mdh1p, which are not as prominent in planktonic cells. Some of these proteins exhibit zinc ion binding activity, which typically involves negatively charged or neutral amino acid residues.^49^ The presence of these proteins on the cell surface may contribute to the overall surface charge of biofilm cells, creating an environment more favorable for electrostatic interactions with MB-P. Further studies investigating the surface protein expression and the roles of these proteins under different growth conditions are required to provide deeper insights into the dynamics of interactions with MB-loaded liposomes.

### Antifungal Action of PDT Mediated by MB-Loaded Liposomes against Planktonic Cells and Biofilms of *C. auris*

After confirming the interaction of liposomal formulations with *C. auris*, we moved on to assess the antifungal effect of PDT using free MB, MB-P, and MB-N formulations. Non-MB-liposome (PL and NL) formulations were also included as control groups. For this, planktonic cultures and biofilms of *C. auris* were exposed to liposomes followed by LED irradiation, and then the antifungal activity of PDT was determined by cell viability via colony-forming units (CFU) counting. Antibiofilm activity was also determined by biomass quantification using a crystal violet (CV) staining assay.

In the results of planktonic cultures, MB-P and MB-N successfully eradicated *C. auris* at a minimum fungicidal concentration (MFC) equivalent to free MB at 0.5 μg/mL, with a significant reduction in CFU values. Conversely, at lower MB concentrations (0.25 μg/mL), both MB-loaded liposome formulations caused greater reductions in *C. auris* burden compared with free MB. Non-MB-loaded liposomes (PL and NL) did not inhibit fungal growth, as expected. Furthermore, no reduction in fungal burden was observed for any treatment group without irradiation, confirming that light alone did not influence fungal viability and light activation is required for antifungal activity of MB and MB-loaded liposomes (Figures 5 and S3).

**Figure 5:**
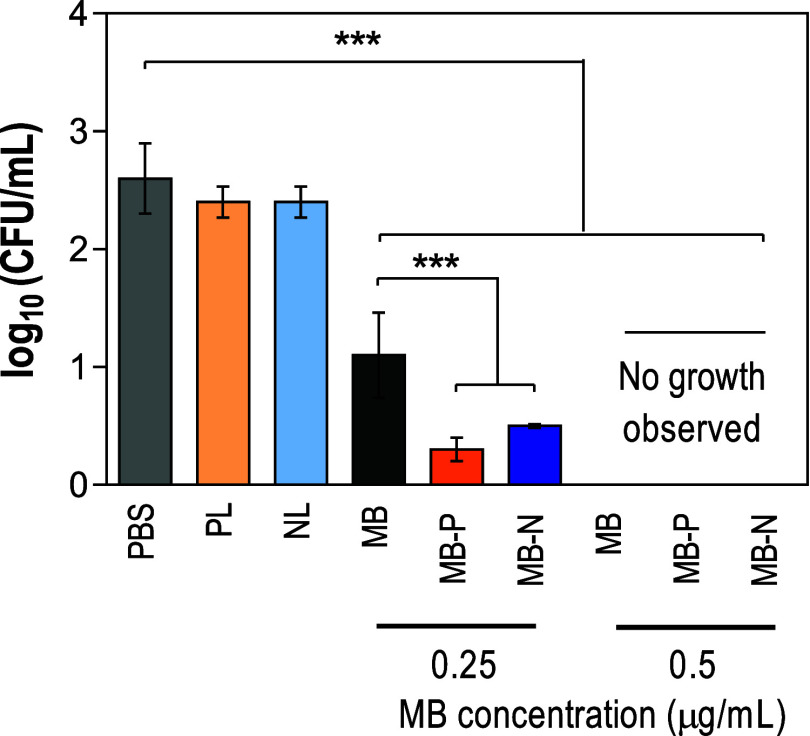
Application of PDT on planktonic cells of *C. auris*. Number of CFU/mL of *C. auris* after 15 min of incubation with liposome formulations (0.25 and 0.5 μg/mL of MB), followed by LED irradiation. PBS: phosphate-buffered saline; PL: positively charged liposomes; NL: negatively charged liposomes; MB: Methylene blue; MB-P: positively charged liposomes encapsulating MB; MB-N: negatively charged liposomes encapsulating MB. The data are represented as mean ± standard deviations (one-way analysis of variance (ANOVA) with Tukey’s posthoc analysis, *n* = 5, α = 0.05, *p ≤ 0.05, **p ≤ 0.01, and ***p ≤ 0.001).

When PDT was applied to *C. auris* biofilms, both MB-P and MB-N liposome formulations significantly reduced the fungal burden at concentrations equivalent to free MB (32 μg/mL). Specifically, MB-P and MB-N resulted in a ∼4.8 and a ∼5.5 log (CFU/mL) reduction, respectively, compared to the control group treated with PBS. In contrast, free MB only resulted in a 1.4 log (CFU/mL) reduction (Figure 6A). Additionally, a significant decrease in *C. auris* biofilm biomass was observed after PDT mediated by MB-loaded liposomes compared to that of biofilms treated with free MB and untreated controls. PDT mediated by MB-P and MB-N led to ∼37 and ∼26% reductions, respectively, while free MB only achieved a ∼3% reduction in biofilm biomass compared to the untreated control (Figure 6B). While not statistically significant, MB-P showed a slightly superior effectiveness in CFU and biomass reduction compared to MB-N. These observations are consistent with CLSM results, indicating that MB-P demonstrated superior penetration of MB within biofilm. Both non-MB-loaded liposomes (PL and NL), as well as treatments without irradiation (Figure S4), did not demonstrate any antibiofilm activity, as expected.

**Figure 6:**
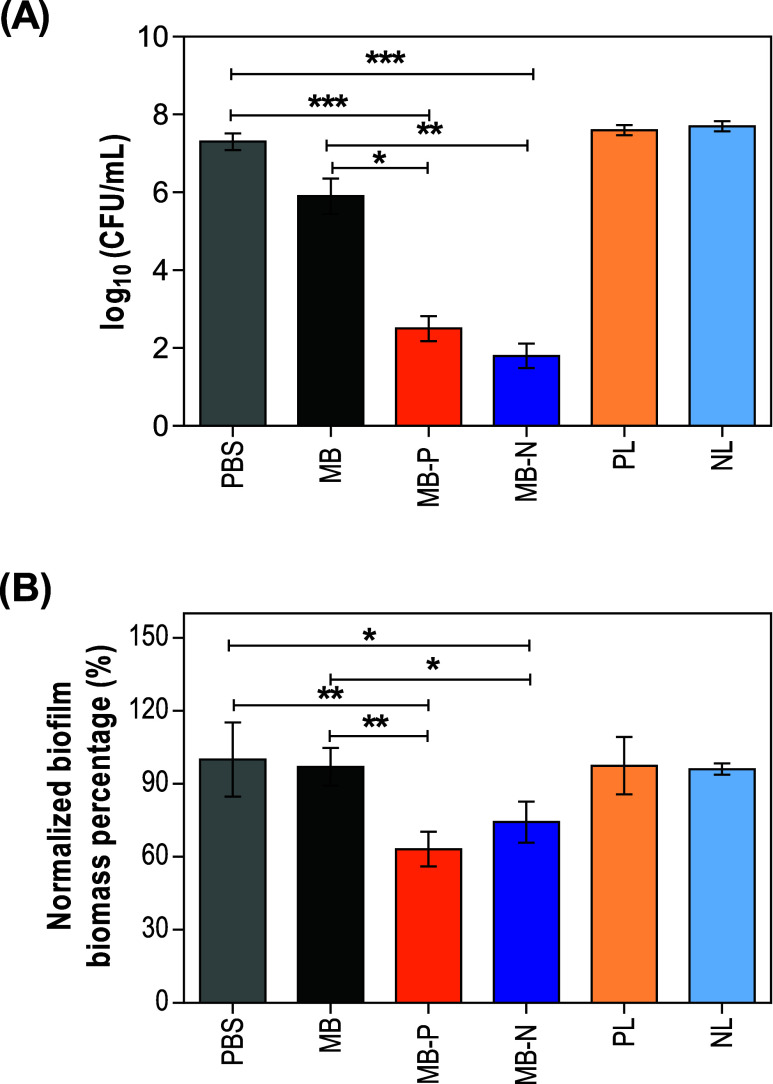
Application of PDT on *C. auris* biofilms. (A) CFU counts of *C. auris* biofilms after a 15 min incubation with liposome formulations (32 μg/mL of MB), followed by LED irradiation. MB-loaded liposomes significantly reduced fungal burden more effectively compared to free MB. The data are represented as mean ± standard deviations (one-way analysis of variance (ANOVA) with Tukey’s posthoc analysis, *n* = 5, α = 0.05, *p ≤ 0.05, **p ≤ 0.01, and ***p ≤ 0.001). (B) Biofilm biomass percentage of *C. auris* post incubation with liposome formulations for 15 min followed by LED irradiation. MB-loaded liposomes significantly reduced biofilm biomass compared with free MB and untreated controls. Data are represented as mean ± standard deviations (one-way analysis of variance (ANOVA) with Tukey’s posthoc analysis, *n* = 5, α = 0.05, *p ≤ 0.05 and **p ≤ 0.01). PBS: phosphate-buffered saline; PL: positively charged liposomes; NL: negatively charged liposomes; MB: Methylene blue; MB-P: positively charged liposomes encapsulating MB; MB-N: negatively charged liposomes encapsulating MB.

Taken together, our findings showed that PDT mediated by MB-P and MB-N liposomes had strong antifungal activity against *C. auris*, causing total elimination of planktonic cells and exhibiting antibiofilm activity. Comparing with other studies in the literature, we can suggest that *C. auris* presents susceptibility to liposome-based PDT with results comparable to *C. albicans* and bacteria. Romio et al.^50^ explored the effects of PDT mediated by liposomes loaded with triclosan and acridine orange against *C. albicans*, verifying 40% inhibition of planktonic cells. In relation to bacteria, Miretti et al.^51^ studied the effects of PDT, using Zn-phthalocyanine (ZnPc) liposomes, on nontuberculous mycobacteria. PDT with ZnPc-liposomes achieved reductions varying from 2.9 to 3.6 log_10_ (CFU/mL) for *Mycobacterium fortuitum* and from 2.0 to 2.6 log_10_ (CFU/mL) for *Mycobacterium chelonae*. Free ZnPc showed minor reductions in bacterial viability (∼0.9 log10 CFU/mL), emphasizing the importance of liposome encapsulation in enhancing efficacy. With focus on a Gram-negative bacterium, Afrasiabi et al.^52^ developed a doxycycline-loaded liposome doped with curcumin (NL-Cur + Dox) to combat *Aggregatibacter actinomycetemcomitans*, a key pathogen in periodontitis. NL-Cur + Dox irradiated with LED light reduced bacterial biofilms by 82%, while light irradiation of nonformulated drugs showed reductions of approximately 40%. These authors suggested that liposomes improved the solubility, stability, and delivery of curcumin and doxycycline, facilitating better drug-cell interactions and potentially enhancing penetration and accumulation at the bacterial site.

These results underline the importance of optimizing photosensitizers by using liposomal formulations for antimicrobial PDT applications. Liposomal formulations designed here were able to increase the delivery of MB in biofilms formed in vitro and to improve PDT effectiveness on *C. auris*. However, additional studies in animal models are required to validate their in vivo efficacy and then translate it for clinical studies. As the clinical application of liposome-based PDT is mainly directed at the treatment of cutaneous and mucosal infections, where the light can be easily applied, future studies using wound mouse infection models should be initiated.

### Effects of PDT Mediated by MB-Loaded Liposomes on Reactive Oxygen Species Production in *C. auris* Biofilm

ROS generation is crucial for effective PDT approaches, as ROS are responsible for causing oxidative damage to microbial cells, leading to cell death.^53^ ROS target cellular components, particularly the cell membrane, causing lipid peroxidation, protein oxidation, and nucleic acid damage.^54,55^ In the PDT process, the photosensitizer dictates the efficiency of ROS production, and then ROS quantification is a prime target for the optimization of drug delivery systems as liposomes.^56^ To confirm the PDT effect of MB-P and MB-N formulations, we assessed the quantification of ROS production in *C. auris* biofilms after exposure to MB-loaded liposomes and LED irradiation. The ROS yield was monitored by using a DCFH-DA probe.

All experiments included the following groups: empty liposomes (PL and NL), MB-loaded liposomes, PBS only, and hydrogen peroxide (positive control). Each condition was tested both with (Figure 7) and without (Figure S5) light irradiation. No increased ROS generation was detected in empty liposomes or PBS under either condition, confirming that the nanocarriers themselves do not produce ROS or interfere with the assay. In contrast, hydrogen peroxide and MB-loaded liposomes under irradiation showed significant ROS production. More specifically, under irradiation, MB-P increased ROS production by ∼2.6× and MB-N by ∼2.2×, in contrast with the significantly lower ROS generation observed with free MB at 32 μg/mL.

**Figure 7:**
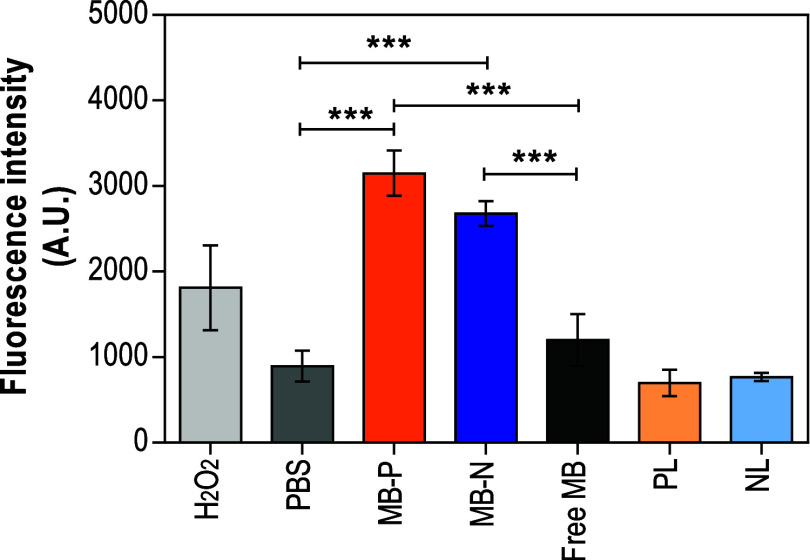
ROS production in *C. auris* biofilm after 15 min of incubation with liposome formulations or free MB (32 μg/mL) followed by light irradiation (PDT). PBS: phosphate-buffered saline; PL: positively charged liposomes; NL: negatively charged liposomes; MB: Methylene blue; MB-P: positively charged liposomes encapsulating MB; MB-N: negatively charged liposomes encapsulating MB. Data are presented as mean ± standard deviations. Statistical analysis was performed using a one-way analysis of variance (ANOVA) with Tukey’s posthoc analysis (*n* = 5, α = 0.05). Significance levels are denoted as ***p ≤ 0.001.

Therefore, greater oxidative damage was generated using MB-loaded liposomes on *C. auris* biofilms compared with free MB, aligning with the CFU count and biomass results. These findings further underscore the relationship between the enhanced MB delivery and increased ROS production within biofilms. Wu et al.^13^ also developed liposomes using a zwitterionic polymer–lipid (poly­(12-(methacryloyloxy)­dodecyl phosphorylcholine)) to encapsulate MB; however, their study was focused on antitumoral photodynamic therapy. These authors employed the DCFH-DA assay to quantify the ROS production in breast cancer cells treated with MB-loaded liposomes and compared it to that of free MB. A strong green fluorescence was observed in cancer cells treated with MB-liposomes after light exposure. The fluorescence intensity in MB-liposome-treated cells was significantly higher than that in cells treated with free MB under the same conditions. According to the authors, improved intracellular ROS generation was attributed to the enhanced cellular uptake of MB when encapsulated in liposomes. The liposomal delivery system facilitated better membrane penetration and a higher intracellular accumulation of MB.

For antimicrobial PDT, in addition to targeting cellular sites, the ROS generated should also target biofilm components. It has been demonstrated that ROS produced after PDT can attack proteins, lipids, and nucleic acids present within the biofilm matrix, contributing to disrupting the extracellular matrix and disaggregating the biofilms.^57^ However, biofilms are more resistant to damage by ROS than planktonic cells. Naturally, the biofilm environment suffers from the accumulation of oxidant agents produced endogenously by the aerobic metabolism of microorganisms and by the mammalian immune system. To defend themselves from ROS damage, biofilms develop protective mechanisms, such as an increased production of specific detoxifying enzymes like catalases and superoxide dismutases (SODs). It is known that there is an intricate interplay between the response to oxidative stress and biofilm formation stages, and many other resistance mechanisms can exist behind the action of detoxifying enzymes.^58^ There is evidence that a common mechanism for oxidative stress resistance and multicellular behavior exists in *C. albicans* biofilms, since cell adhesion, biofilm formation, and oxidative stress resistance are influenced by a common factor, the cell wall protein Hwp2p.^59^

Therefore, for the effectiveness of PDT on biofilms, photosensitizers must be designed to generate high levels of intra- and extracellular reactive oxygen species (iROS and eROS), and MB-loaded liposomes developed in our study reinforce the notion that optimized photosensitizer delivery can improve PDT outcomes against biofilms. Seeking to increase the ROS production, recent studies have also focused on synergistic approaches, integrating liposome-based PDT with other ROS generation strategies, such as sonodynamic therapy (SDT) and photothermal therapy (PTT).^56,60,61^

### Evaluation of Possible Cytotoxic Effects of MB-Loaded Liposomes on Mammalian Cells

For a safe clinical application, liposomal formulations must combine antimicrobial efficacy without toxicity on host cells.^62,63^ Thus, in this study, we measured the cell viability of NIH/3T3 fibroblasts after contact with MB-loaded liposomes for a period of 15 min in the dark, using a CCK-8 assay. Fibroblasts were chosen as a model cell line, as they are one of the main cell types found in the skin and in localized *C. auris* infections (e.g., burn wounds, skin infections).

At a concentration of 32 μg/mL, free MB significantly reduced the metabolic activity of fibroblasts (∼58%) compared to that of untreated controls; both liposome formulations (MB-P and MB-N) maintained fibroblast viability at ∼80%. These findings suggest that liposomes provided a protective effect of MB on fibroblasts. As expected, the lowest cell viability was observed in the 10% (v/v) DMSO control group (Figure 8).

**Figure 8:**
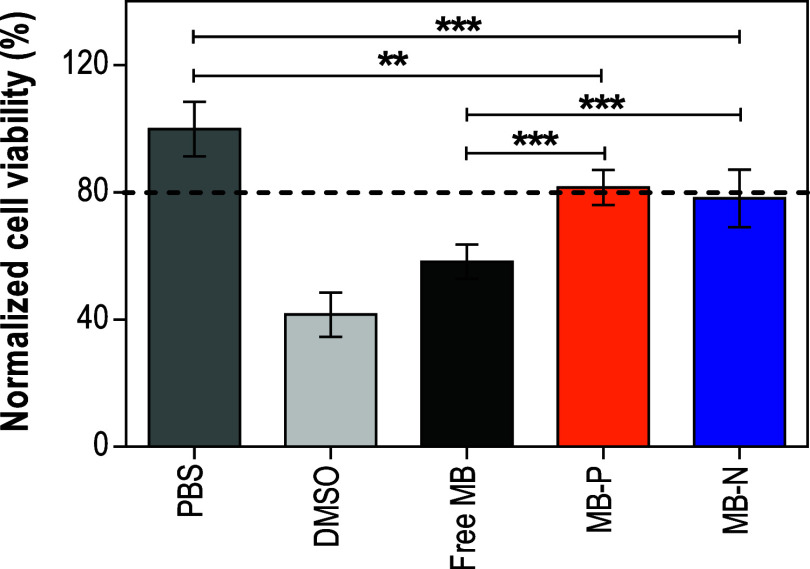
Cell viability of NIH/3T3 fibroblasts following 15 min incubation in the dark with liposome formulations and free MB, as measured by a CCK-8 assay. PBS: phosphate-buffered saline; PL: positively charged liposomes; NL: negatively charged liposomes; MB: methylene blue; MB-P: positively charged liposomes encapsulating MB; MB-N: negatively charged liposomes encapsulating MB. Data are presented as mean ± standard deviations (one-way analysis of variance (ANOVA) with Tukey’s posthoc analysis, *n* = 5, α = 0.05, **p ≤ 0.01 and ***p ≤ 0.001).

It has been seen that MB can influence cell proliferation and cell signaling and induce apoptosis in a dose and time-dependent manner. Moreover, systemic administration of MB has been associated with adverse effects such as hemolysis, methemoglobinemia, and nausea.^31^ Our findings indicate that MB-loaded liposome formulations can be used to reduce MB toxicity toward fibroblasts at therapeutic concentrations (e.g., 32 μg/mL). This observed phenomenon may be attributed to the liposome drug delivery mechanism, which probably caused a gradual release of MB, decreasing its direct contact with fibroblasts compared to free MB. In addition, in the context of PDT, it has been proposed that when liposomes are exposed to light, their thermosensitive lipid composition, such as DPPC, induces degradation and subsequent release of their inner content.^64^ Consequently, a certain proportion of liposomes may release their content only in response to light exposure. In the absence of light, these liposomes may play a protective role. Overall, this suggests a promising avenue for the development of safe and effective antifungal PDT by using MB-loaded liposomes.

Similar results were reported by Afrasiabi et al.,^52^ who designed doxycycline-loaded liposomes doped with curcumin (NL-Cur + Dox) against *A. actinomycetemcomitans*, testing their possible toxic effects on Human Gingival Fibroblasts (HGFs) and red blood cells. The cytotoxicity and hemocompatibility of NL-Cur + Dox were compared to free doxycycline (Dox) and curcumin (Cur). NL-Cur + Dox and NL-Cur maintained cell viability up to 90%, while treatment with free Dox reduced cell viability significantly, indicating that liposomal encapsulation protected HGFs from the cytotoxic effects of free Dox. NL-Cur + Dox exhibited low hemolysis (<5%), comparable to the negative control (PBS). Red blood cells (RBCs) treated with NL-Cur + Dox showed no morphological changes, further confirming their safety for systemic use.

By combining our results with data from the literature, we can confirm that the encapsulation of photosensitizers in liposomes not only improves therapeutic efficacy but also minimizes cytotoxic effects on mammalian cells, increasing the selectivity of PDT.

## Conclusion

In this in vitro study, the designed liposomes using saturated lipids were able to encapsulate MB photosensitizers and provide an efficient delivery to *C. auris* cells and biofilms. Among the formulations developed, MB-Positive liposomes had a greater interaction with *C. auris* biofilms than MB-Negative liposomes, which provided a higher penetration of MB within the biofilms. When PDT was applied, both MB-loaded liposomes caused an eradication of *C. auris* planktonic cells and an inhibition of biofilms compared with free MB. MB-P liposomes showed a slightly superior effectiveness in reducing the number of viable *Candida* cells and total biomass of biofilms. The decrease of biofilms after PDT mediated by MB-loaded liposomes was accompanied by an increase in the ROS production in biofilms. Moreover, MB-loaded liposomes demonstrated biocompatibility with mammalian fibroblasts. This study is the first to demonstrate the in vitro effectiveness of PDT using PS-loaded liposomes to control *C. auris* biofilms, providing support for further in vivo studies that can validate its efficacy and safety for the treatment of *C. auris* infections.

## Methods

### Materials

All lipids (1,2-dipalmitoyl-*sn*-glycero-3-phosphocholine (DPPC), cholesterol (CHOL), 1,2-dipalmitoyl-*sn*-glycero-3-phospho-(1′-rac-glycerol) sodium salt (DPPG), and dimethyldioctadecylammonium bromide (DDAB)) were purchased from Avanti Polar Lipids, Inc. (Alabaster, Alabama). Chloroform, methanol, sodium hydroxide, phosphate-buffered saline (PBS), calcofluor white (CFW) stain, methylene blue (MB), crystal violet (CV) stain, Yeast Peptone Dextrose (YPD) medium, Yeast Nitrogen Base (YNB) medium, d-(+)-Glucose, ≥99.5% (GC), 2′,7′-dichlorodihydrofluorescein diacetate (H2DCFDA), Vivaspin 20 centrifugal concentrator, MWCO 300 kDa (Sartorius, Hannover, Germany) and dimethyl sulfoxide (DMSO) were all purchased from Sigma-Aldrich (St. Louis, Missouri). UV-transparent disposable cuvettes were purchased from Sarstedt (Nümbrecht, Germany). Capillary zeta cells were purchased from Malvern Panalytical (Westborough, Massachusetts). Sabouraud dextrose (SD) agar was purchased from Research Products International (Mount Prospect, Illinois). Hydrogen peroxide (30% v/v) was purchased from Fisher Chemical (Insert City, New Jersey). Polycarbonate membranes (0.2 μm) and Dulbecco’s Modified Eagle Medium (DMEM) were purchased from Cytiva Life Sciences (Marlborough, Massachusetts). *C. auris* B11220 and NIH 3T3 fibroblast cells were purchased from the American Type Culture Collection (ATCC, Manassas, Virginia). Lab-Tek 2-well chamber slides were purchased from Thermo Fisher Scientific (Waltham, Massachusetts). The Cell Counting Kit-8 (CCK-8) assay kit was purchased from APExBIO Technology, LLC (Houston, Texas). Ultrapure water was utilized for all experiments, as needed (18.2 MΩ·cm Milli-Q, Millipore Sigma, Billerica, Massachusetts).

### Fabrication of Liposomes

DPPC, CHOL, and DDAB stocks were made in chloroform at a concentration of 25 mg/mL, while DPPG stocks were made in a chloroform: methanol solution (5:1 v/v at a concentration of 2 mg/mL). All lipid stocks were stored at −20 °C prior to use. A fresh aliquot of MB was prepared in PBS (1 mg/mL) each time before use. MB-P and MB-N-charged MB-loaded liposomes were fabricated by using DDAB and DPPG, respectively. To fabricate the MB-loaded liposomes, lipids were added into glass vials in the desired ratios (Table 2); CHOL was added to achieve a final concentration of 20% (v/v) in all formulations. Chloroform and/or methanol were removed using a rotary evaporator in a 40 °C water bath (Rotavapor R-300, Buchi, Sankt Gallen, Switzerland). The resulting dry lipid film was stored overnight in a desiccator. The dry lipid film was then rehydrated at 69 °C in MB solution to a final lipid concentration of 10 mg/mL. Rehydrated samples were then vortexed, sonicated at 69 °C for 30 min, and extruded through 200 nm polycarbonate membrane for a total of 10 extrusions using a Liposofast LF-50 extruder (Avestin, Ottawa, Canada) at 70 °C. Liposome formulations were purified to remove nonencapsulated MB by ultracentrifugation with a Vivaspin 20 centrifugal concentrator with 300 kDa pore size at 4000*g* for 1 h. Non-MB-loaded liposomes were rehydrated with PBS without MB and fabricated as described above. All liposomal formulations were stored at 4 °C until further use.

**Table 2:** Liposome Composition of Positively (MB-P) and Negatively (MB-N) Charged Methylene Blue (MB) Loaded Liposomes

	components (w/w)				
liposomes	DPPC	DPPG	DDAB	CHOL	MB in preparation solution (mg/mL)
MB-P	3		1	1	1
MB-N	3	1		1	1

### Characterization of Liposomes According to Physical Properties, Encapsulation Efficiency, and Drug Loading Capacity

To obtain the hydrodynamic diameter and polydispersity index (PDI) of each liposome formulation, dynamic light scattering (DLS) measurements were performed on a Zetasizer Nano ZS90 instrument (Malvern Panalytical, Westborough, Massachusetts). An aliquot of 50 μL of each sample was added to a UV-transparent disposable cuvette (Sarstedt, Nümbrecht, Germany). Three measurements were obtained per sample. The ζ-potential of each formulation was obtained by making a 10-fold dilution of each liposomal formulation in PBS; measurements were collected using the Zetasizer Nano ZS90. The encapsulation efficiency (EE%) was assessed via absorbance measurements. Briefly, liposomes were lysed with 70% (v/v) methanol in a 1:1 liposome:methanol ratio (v/v). Lysed samples were transferred to a 96-well microtiter plate, and the absorbance of each well was collected from 500 to 800 nm using a Cytation 3 plate reader (BioTek, Winooski, Vermont). Using a standard curve of free MB prepared in PBS and 70% (v/v) methanol, the EE% was calculated using eq 1. To determine the drug loading (DL%) capacity of each formulation, liposomes were prepared in a 1:1 (v/v) solution of PBS and lyophilized to obtain the liposome dry weight. The MB concentration was measured as described above and the DL% was calculated using eq 2.

1 $$\mathrm{EE}\%=\frac{\mathrm{total}\;\mathrm{amount}\;\mathrm{of}\;\mathrm{MB}\;\mathrm{in}\;\mathrm{liposome}\;\left(\mathrm{mg}\right)}{\mathrm{total}\;\mathrm{amount}\;\mathrm{of}\;\mathrm{MB}\;\mathrm{added}\;\mathrm{initially}\;\left(\mathrm{mg}\right)}\times 100$$

2 $$DL\%=\frac{total\;amount\;of\;MB\;in\;liposome\;\left(mg\right)}{liposome\;dry\;mass\;\left(mg\right)}\times 100$$

### MB-Loaded Liposome Interaction with *C. auris* via Flow Cytometry

To verify interaction between liposomes and fungal cells, flow cytometry was used as described by LaMastro et al.,^21^ with modifications. An overnight culture of *C. auris* was prepared by selecting five colonies from an SD agar plate and incubating the culture in 5 mL of YPD media for 18 h at 37 °C and shaking at 120 rpm. The overnight culture was adjusted to 10^8^ CFU/mL (OD530:1.258) in YNB media containing 100 μM of glucose. Next, 10 μL of adjusted *C. auris* culture was treated with either MB-P or MB-N liposomes in PBS (140 μL/well) in a 96-well plate, achieving a 1.4:0.1:0.5 (v/v/v) ratio, and incubated for 15 min at 37 °C, shaken at 120 rpm, and protected from light; PBS (50 μL/well) was added instead of MB-loaded liposomes as a control. As MB is a fluorescent dye with λ_ex_ 665 nm and λ_em_ 686 nm,^65^ no addition of other fluorescent dye was needed. Samples were then immediately analyzed by flow cytometry. At least 50,000 events per sample were collected. Data was analyzed with FlowJo Single Cell Analysis Software, v10 (Becton, Dickinson and Co. Biosciences, Franklin Lakes, New Jersey). Gating was performed by utilizing samples containing cells only (no liposomes) and liposomes alone (no cells).

### MB-Loaded Liposome Interaction with *C. auris* via Confocal Laser Scanning Microscopy

The interaction between the MB-loaded liposomes (MB-P and MB-N) and planktonic *C. auris* was examined by using confocal laser scanning microscopy (CLSM). Briefly, fungal cells were exposed to free MB, MB-P, MB-N, or PBS for 15 min at 37 °C and shaking at 120 rpm protected from light. After incubation, *C. auris* were stained with 1 μg/mL of CFW stain and 10 mM sodium hydroxide for 1 min protected from light. Next, 10 μL of the suspension was placed on a glass slide, covered with a glass coverslip, and imaged.

The MB interaction and penetration into *C. auris* biofilms were also examined using CSLM. Briefly, mature *C. auris* biofilms, grown for 24 h at 37 °C, were washed twice with PBS to remove unattached cells. MB-N, MB-P, free MB, or PBS were added, and plates were incubated for 15 min in the dark at 37 °C. After this, wells were washed gently with PBS. CFW was added to each well and incubated for 2 min at room temperature, protected from light. The supernatant from each well was removed, and the biofilm was washed with PBS gently. Biofilms were then imaged, and normalized MB fluorescence intensity was calculated using eq 3.

3 normalizedMBfluorescenceintensity(%)={[sample(intensity/area)−negativecontrol(intensity/area)]/[positivecontrol(intensity/area)−negativecontrol(intensity/area)]}×100

All CLSM images were obtained by using an Apo LWD 20×/1.10 water immersion objective and an A1R confocal laser microscope (Nikon Instruments, Inc., Melville, New York).

### Activity of PDT Mediated by Liposomal Formulations against Planktonic Cells of *C. auris*

After 24 h growth of *C. auris* in YPD medium, *C. auris* was centrifuged and washed twice with PBS. The fungal suspension was adjusted to 10^3^ CFU/mL in PBS. Then, in a 96-well microtiter, 100 μL/well of free MB, MB-P, MB-N, or PBS was added; free MB was freshly dissolved in sterile PBS, filtered with a 0.22 μm membrane, and protected from light. Next, 100 μL of a fungal suspension, adjusted to 10^3^ CFU/mL, was added to each well. Plates were then protected from light and incubated at 37 °C, 120 rpm, for 15 min (preirradiation time). Then, samples were either irradiated for 712 s or not subjected to irradiation as a control. A light-emitting diode (LED, BlackBox smart, Biolambda, Brazil) emitting a wavelength at 660 nm (visible red) was used as the light source. The irradiance used was 40.11 mW/cm^2^, and the radiant exposure was 28.56 J/cm^2^. Each well was serially diluted, and 5 μL of each experimental group was plated on SD agar and incubated at 37 °C for 24 h. The fungal burden was then quantified via colony-forming units (CFU) counting. *C. auris* cells treated with just PBS or non-MB-containing liposomes were used as the controls.

### Activity of PDT Mediated with MB-Loaded Liposomes on *C. auris* Biofilms

Biofilm susceptibility was assessed against intermediate-stage (24 h) monotypic *C. auris* biofilms. Initially, 200 μL/well of fungal suspension, adjusted to 10^6^ CFU/mL in YNB medium, was added to a 96-well microtiter plate and incubated for 90 min at 37 °C and 120 rpm to allow for initial adhesion. The supernatant was then aspirated, and the biofilm was washed twice with PBS to remove nonadherent cells. YNB medium (200 μL) was added to each well and the plates incubated at 37 °C, 120 rpm. After 24 h, the supernatant was removed, and the wells were washed twice with PBS. Biofilms were then treated with free MB, MB-loaded liposomes, or PBS at the desired concentration for 15 min (preirradiation time) at 37 °C, 120 rpm, protected from light. After this, samples were irradiated (L+) as described above, while control samples (L−) were kept protected from light. Biofilms were then disrupted with vigorous pipetting, and CFU was quantified by plating 5 μL of the dilutions of each well on SD agar. Biofilms treated with PBS and non-MB-loaded liposomes were included as controls.

To quantify the biofilm biomass after PDT, monotypic *C. auris* biofilms were prepared, as described in the section “In vitro activity of PDT mediated with MB-loaded liposomes on *C. auris* biofilms.” Following treatment and subsequent irradiation, biofilms were gently washed twice with PBS, air-dried for 45 min, and stained with a 0.1% (w/v) CV solution (100 μL/well) for 15 min. The stained biofilms were then washed twice with PBS. CV stain was eluted with 100 μL/well of 100% methanol. The absorbance was measured at 540 nm and the normalized biofilm biomass was calculated as follows (eq 4)­

4 $$biofilm\;biomass\;\left(\%\right)=\frac{sample\;\left({Abs}_{540}\right)-negative\;control\;\left({Abs}_{540}\right)}{positive\;control\;\left({Abs}_{540}\right)-negative\;control\;\left({Abs}_{540}\right)}\times 100$$

### Reactive Oxygen Species Production in Biofilms Treated with PDT Mediated by MB-Loaded Liposomes

Reactive oxygen species (ROS) production in biofilms after PDT was monitored by the conversion of nonfluorescent probe 2′,7′-dichlorodihydrofluorescein diacetate (H_2_DCFDA) to fluorescent 2′,7′-dichlorofluorescein (DCF). Biofilms were prepared as described previously and treated with PBS, free MB, MB-P, MB-N, NL, or PL. Hydrogen peroxide (H_2_O_2_) at a final concentration of 3 mM was included as a positive control. The plates were wrapped in aluminum foil, incubated for 15 min at 37 °C and 120 rpm, and then irradiated as previously described or kept in the dark. Then, wells were washed twice, and 100 μL of the H_2_DCFDA at a final concentration of 1 μM was added and maintained for 30 min at 37 °C, and 120 rpm. All samples were transferred to a black 96-well plate without surface treatment. Fluorescence was measured in the spectral range of an excitation of 485 nm and emission of 538 nm.

### Analysis of Cytotoxicity of MB-Loaded Liposome on Mammalian Cells

Cell viability of fibroblasts after exposure to MB-P and MB-N was evaluated using a CCK-8 cell viability assay. In a 96-well plate, fibroblast was seeded at a density of 10,000 cells/cm^2^ in DMEM and incubated for 24 h at 37 °C and 5% CO_2_. Next, fibroblasts were treated with 10% (v/v) MB, liposomes, or PBS for 15 min at 37 °C and 5% CO_2_ at the same concentration used for biofilm studies. As a positive cytotoxic control of 10% (v/v) DMSO was included. After the incubation period, the media was replaced with 10% (v/v) CCK-8 solution prepared in DMEM, and plates were incubated for 2 h at 37 °C and 5% CO_2_. Absorbance was measured at 450 nm. Cell viability was calculated using eq 5.

5 $$normalized\;cell\;viability\;\left(\%\right)=\frac{sample\;\left(\mathrm{Abs}\right)-negative\;control\;\left(Abs\right)}{positive\;control\;\left(Abs\right)-negative\;control\;\left(Abs\right)}\times 100$$

### Statistical Analysis

Statistical significance was analyzed using GraphPad Prism 5 with one-way analysis of variance (ANOVA) with posthoc Tuckey’s test where applicable (α = 0.05; *p* < 0.05 was considered statistically significant; *p < 0.05, **p < 0.01, ***p < 0.001, ****p < 0.0001). All experiments were conducted in triplicate at a minimum.

## Supplementary Material



## Notes

Additional information on MB-loaded liposome fabrication using a schematic representation (Figure S1); additional information about flow cytometry gating strategy for *C. auris*-liposome interaction studies (Figure S2); results from CFU counts of *C. auris* B11220 following 15 min incubation with liposome formulations without irradiation (Figure S3); results of antibiofilm activity of MB-loaded liposomes against *C. auris* biofilms without irradiation (Figure S4); results of ROS production in *C. auris* B11220 biofilms after 15 min incubation with liposome formulations without irradiation (Figure S5) (PDF)
