# Supplementary material for: Methylene Blue-Loaded Liposomal Nanocarriers Enhance the Efficacy of Photodynamic Therapy against Candida auris Biofilm
Source: ACS Infect Dis. 2025 Dec 25;12(1):425–37. doi: 10.1021/acsinfecdis.5c00941 (PMC12797236; doi:10.1021/acsinfecdis.5c00941)
Supplement: Supplementary file 1 [file id5c00941_si_001.pdf]

**Supporting Information for Publication**

# Methylene Blue-Loaded Liposomal Nanocarriers Enhance the Efficacy of Photodynamic Therapy Against *Candida auris* Biofilm

Patricia Michelle Nagai de Lima[1,2*], Akram Abbasi, Ph.D.[1], Veronica LaMastro[1], Juliana Campos Junqueira, Ph.D.[2*], and Anita Shukla, Ph.D.[1*]

1. Center for Biomedical Engineering, School of Engineering, Brown University, Providence, RI, USA

2. Institute of Science and Technology, São Paulo State University (UNESP), São Paulo, Brazil

*To whom Correspondence should be addressed:

*Patricia Michelle Nagai de Lima: patricia.nagai@unesp.br

*Juliana Campos Junqueira: juliana.junqueira@unesp.br

*Anita Shukla: anita_shukla@brown.edu

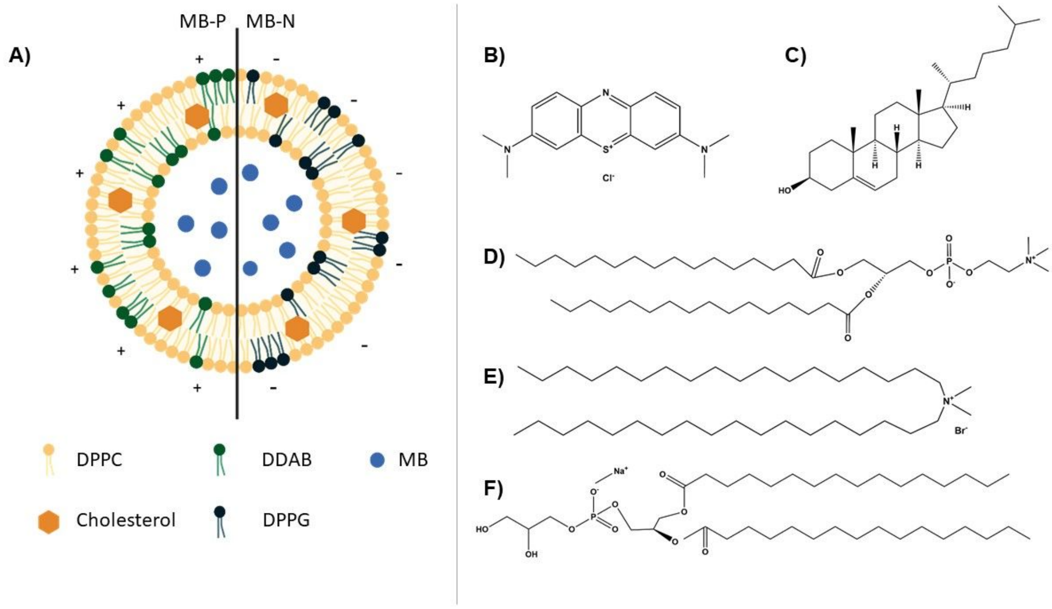


**Figure S1:** Representative schematic of liposome design, with (A) demonstrating methylene blue (MB) encapsulation within positively charged (MB-P) and negatively charged liposomes (MB-N) fabricated with DPPC, cholesterol, DDAB (for MB-P), and DPPG (for MB-N). Chemical structures of the liposome components: (B) Methylene blue; (C) cholesterol; (D) 1,2-dipalmitoyl-sn-glycero-3-phosphocholine; (E) dimethyldioctadecylammonium bromide; and (F) 1,2-dipalmitoyl-sn-glycero-3-phospho-(1'-rac-glycerol) sodium salt.

All flow cytometry samples were analyzed using FlowJo software (v 10). In each experiment, a single cell type was utilized, and forward scatter (FSC) and side scatter (SSC) profiles were employed to accurately gate each sample. In brief, a gate was established around *C. auris*, with exclusion of cell debris and/or dead cells. Subsequently, the population of interest was assessed using FSC Area (FSC-A) versus FSC Height (FSC-H) and SSC-A versus SSC-H. To eliminate doublets, only events falling along an approximately straight line to the densest event area (indicated by red/yellow/green hot spots) were selected for analysis. Lastly, the MB-positive gate was set using the negative cell control group (*C. auris* with no liposome treatment) in a single-parameter histogram.

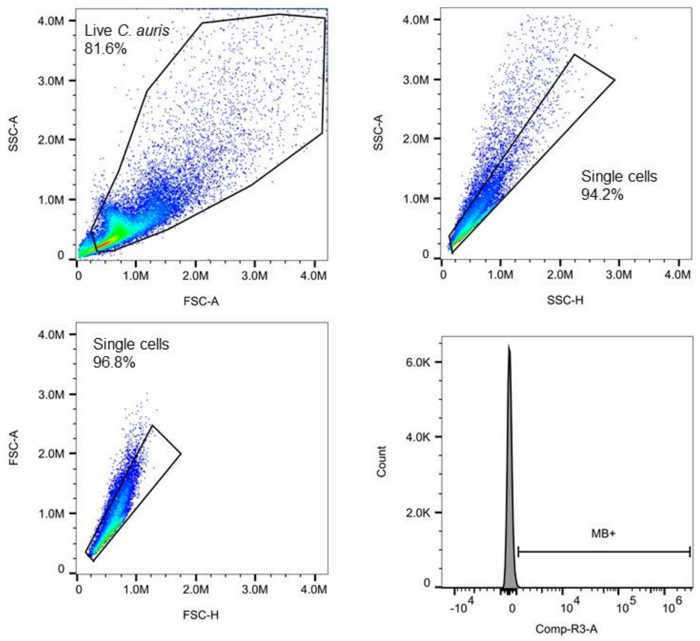


**Figure S2:** Flow cytometry gating strategy for *C. auris*-liposome interaction studies. Cellular debris/dead cells were gated out based on Forward Scatter Area (FSC-A)/Side Scatter Area (SSC-A). A single parameter histogram was used to set an appropriate MB-

positive gate using the negative control containing no liposomes, where the frequency of non-specific MB-signal was set below 1%.

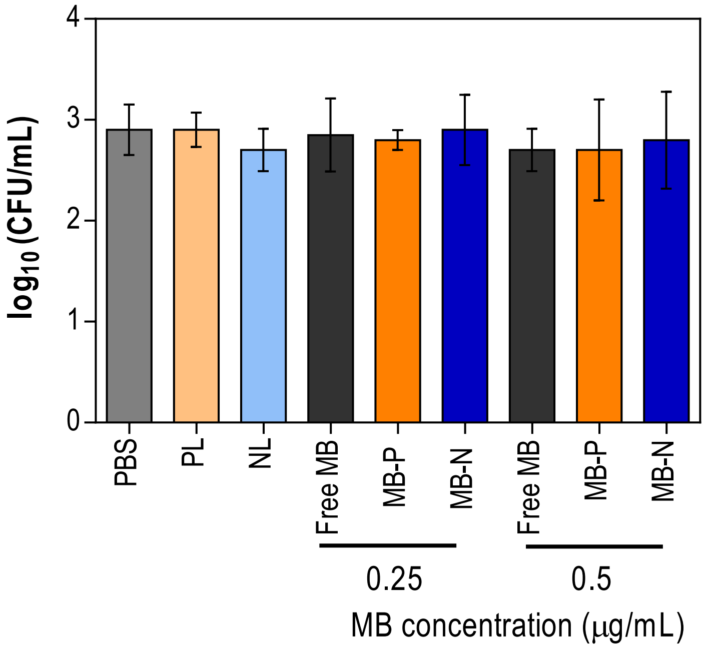


**Figure S3:** CFU counts of *C. auris* following a 15 min. incubation with liposome formulations, without irradiation. PBS: phosphate buffered saline; PL: positively charged liposomes; NL negatively charged liposomes; MB: Methylene blue; MB-P: positively charged liposomes encapsulating MB; MB-N: negatively charged liposomes encapsulating MB. The data are represented as mean ± standard deviations (one-way analysis of variance (ANOVA) with Tukey's post-hoc analysis, n=5, α = 0.05).

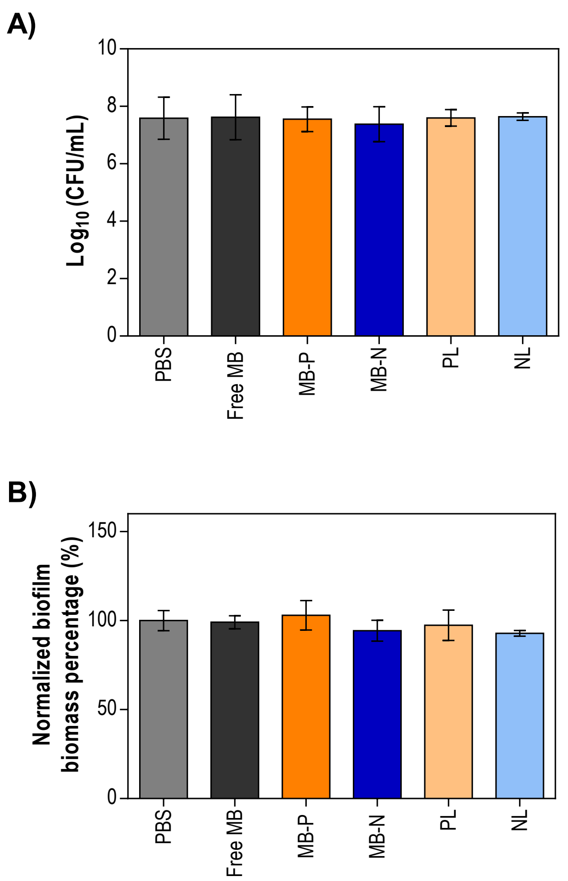


**Figure S4:** Antibiofilm activity of MB-loaded liposomes against *C. auris* biofilms. PBS: phosphate buffered saline; PL: positively charged liposomes; NL negatively charged liposomes; MB: Methylene blue; MB-P: positively charged liposomes encapsulating MB; MB-N: negatively charged liposomes encapsulating MB. A) CFU counts of *C. auris* biofilms following a 15 min incubation with liposome formulations, without irradiation. The data are represented as mean ± standard deviations (one-way analysis of variance (ANOVA) with Tukey's post-hoc analysis, n=5, $\alpha = 0.05$. B) Biofilm biomass percentage of *C. auris* B11220 post incubation with liposome formulations for 15 min, without irradiation. Data are represented as mean ± standard deviations (one-way analysis of variance (ANOVA) with Tukey's post-hoc analysis, n=5, $\alpha = 0.05$.

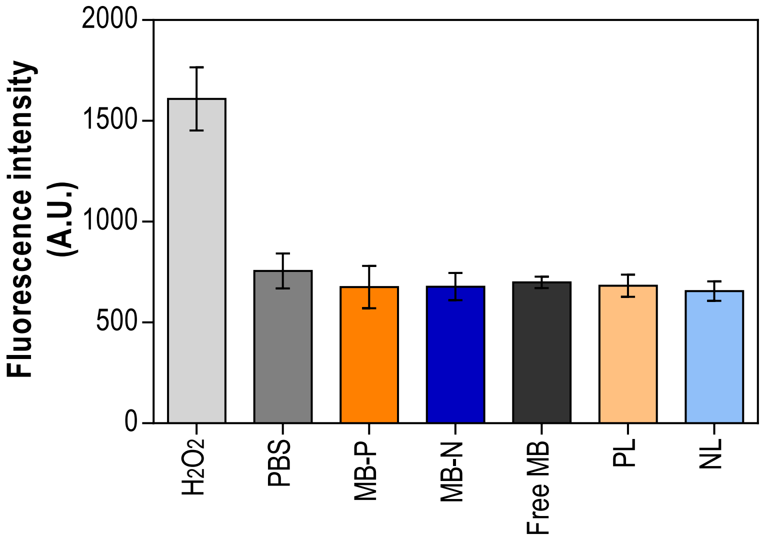


**Figure S5:** ROS production in *C. auris* biofilms after 15 min of incubation with liposome formulations, without irradiation. PBS: phosphate buffered saline; PL: positively charged liposomes; NL negatively charged liposomes; MB: Methylene blue; MB-P: positively charged liposomes encapsulating MB; MB-N: negatively charged liposomes encapsulating MB. Data are presented as mean ± standard deviations. Statistical analysis was performed using a one-way analysis of variance (ANOVA) with Tukey's post-hoc analysis (n=5, $\alpha = 0.05$).
